# Imported Malaria at a Referral Hospital in Tokyo from 2005 to 2016: Clinical Experience and Challenges in a Non-Endemic Setting

**DOI:** 10.4269/ajtmh.18-0722

**Published:** 2019-01-21

**Authors:** Saho Takaya, Yasuyuki Kato, Yuichi Katanami, Kei Yamamoto, Satoshi Kutsuna, Nozomi Takeshita, Kayoko Hayakawa, Shuzo Kanagawa, Kanako Komaki-Yasuda, Shigeyuki Kano, Norio Ohmagari

**Affiliations:** 1 Disease Control and Prevention Center, National Center for Global Health and Medicine, Shinjuku, Japan;; 2 Department of Tropical Medicine and Malaria, National Center for Global Health and Medicine Research Institute, Shinjuku, Japan

## Abstract

In this study, we reviewed imported malaria cases observed at the National Center for Global Health and Medicine, Tokyo, between 2005 and 2016, to comprehend their demographic and clinical characteristics. Data on 169 cases were used to analyze demographic information; data on 146 cases were used for the analysis of clinical information. The median patients’ age was 34 years, and 79.3% of them were male. The proportion of non-Japanese patients increased and surpassed that of Japanese patients after 2015. In 82.2% of the cases, the region of acquisition was Africa, and *Plasmodium falciparum* was the dominant species (74.0%) followed by *Plasmodium vivax* (15.4%). We observed 19 (18.4%, 19/103) severe falciparum malaria cases. Mefloquine was the most commonly used drug for treatment until the early 2010s; atovaquone/proguanil was the most commonly used after its licensure in 2013. Although none of the patients died, four recrudescence episodes after artemether/lumefantrine (A/L) treatment and one relapse episode were observed. Overall, malaria was diagnosed on median day 4 of illness, and, thereon, treatment was initiated without delay. Diagnosis on day 5 or later was significantly associated with severe disease in Japanese cases (odds ratio = 4.1; 95% CI = 1.2–14.3). We observed a dominance of falciparum malaria, an increase in the number of non-Japanese cases, late treatment failure after A/L treatment, a low relapse rate, and an association between delayed malaria diagnosis and higher disease severity. Pretravel care and early diagnosis are necessary to reduce malaria-related mortality and morbidity in settings such as ours.

## INTRODUCTION

Malaria is the most important parasitic infection in the world. Worldwide, a total of 219 million cases and 435,000 related deaths were estimated in 2017, of which more than 90% occurred in Africa.^1^ Imported malaria is a significant health problem in non-endemic countries. Globally, around 10,000–30,000 cases of imported malaria are reported every year.^2,3^ Malaria is the most commonly diagnosed disease associated with febrile illness among returned travelers, especially those returning from Africa, as per the GeoSentinel surveillance report.^4^

In Japan, indigenous malaria was eradicated in 1961, and, thereafter, only cases of imported malaria have been reported, except for a rare case via platelet transfusion.^5^ The number of malaria cases per year was over 100 in the 1990s^6–8^ and then reduced to 50–70 cases in the 2000s.^9^ *Plasmodium vivax* represented approximately half of all malaria cases in the 1990s; thereon, the proportion of *Plasmodium falciparum* cases started to increase gradually. The case fatality rate due to falciparum malaria was 3.3% nationally in the 1990s, which was higher than the rate in European countries at the time.^7^

Management of imported malaria is a challenge and sporadic fatal cases have been reported in Japan. First, Japanese travelers are not well-informed about travel-related illnesses; Asians are less likely to seek pretravel care than those from Western countries.^10,11^ Second, the lack of experience among health-care providers in Japan is considered to be more significant than that in Europe and North America. The malaria incidence in Japan is approximately one-sixtieth of that in the United Kingdom and France, and one-tenth of that in the United States.^3,12^ The resulting health-care inexperience can lead to delayed diagnoses and ultimately increased mortality risk among patients. Last, antimalarial treatment in Japan is largely limited as the use of many antimalarials is unlicensed in the country. This situation of unavailability has been improving gradually in the last two decades, and mefloquine, atovaquone/proguanil (A/P), and artemether/lumefantrine (A/L) are now officially registered. Any form of artesunate and injectable quinine was not licensed as of 2018. Injectable quinine is provided on a research basis through the Research Group on Chemotherapy of Tropical Diseases.^13^

It is important to understand the current imported malaria situation in Japan. However, no studies in the last decade have focused on the related clinical aspects, except for a recent study which used data from the National Epidemiological Surveillance of Infectious Disease system.^12^ We, therefore, reviewed malaria cases at our medical center from 2005 to 2016, to better understand the clinical presentation and management of imported malaria, and the aforementioned challenges.

## MATERIALS AND METHODS

The National Center for Global Health and Medicine (NCGM), located in central Tokyo, is a member of the GeoSentinel Network.^14^ The NCGM serves as a referral hospital for tropical and infectious diseases, and treats the largest number of malaria cases in the country, all of which are confirmed by the NCGM Research Institute.

Malaria cases seen at the NCGM from January 2005 to December 2016 were identified through a patient database. The malaria-causing species included *P. falciparum*, *P. vivax*, *Plasmodium ovale*, *Plasmodium malariae*, and *Plasmodium knowlesi*. Malaria diagnosis was confirmed by blood film microscopy and/or polymerase chain reaction (PCR) performed by skilled researchers. Rapid diagnostic tests (RDTs) were also routinely performed (BinaxNOW^®^ Malaria [Binax Inc., Scarborough, ME] and SD BIOLINE^®^ Malaria Ag P.f/Pan [Standard Diagnostics Inc., Yongin, Republic of Korea]). When the diagnosis was confirmed in a former medical institution with a referral letter stating the validity of the diagnostic method used, such cases were also included. Patients referred to the NCGM only for the radical treatment of vivax/ovale malaria were included for the analysis of basic demography and primaquine treatment. If patients had recrudescence or relapse, only the initial episode was included.

The following basic demographic parameters were reviewed: age, gender, nationality, residency in Japan, region of acquisition, travel reason and duration, time from return to/arrival in Japan to onset, malaria chemoprophylaxis, and *Plasmodium* species. The following clinical parameters were reviewed: comorbidities, symptoms, diagnostic method (RDT, thin blood smear, and PCR), parasite count, severity, antimalarial regimen and its adverse events, hospitalization duration, treatment outcome, and care-seeking behavior. The regions of acquisition were classified according to the United Nation geoscheme.^15^ Travel reasons were modified based on the definitions in the GeoSentinel surveillance^4^ and classified into nine categories: tourism, visiting friends and relatives (VFR), business, missionary, student, international cooperation (IC) activities, volunteer, research, and visiting Japan. International cooperation activities include those in nongovernmental organizations and administrative agencies. Although malaria severity was assessed clinically by attending doctors based on the widely recognized criteria, the epidemiological and research definition of severe falciparum malaria as put forth by the WHO^16^ was applied in this study. The risk factors for severe malaria were analyzed. Antimalarials provided through the Research Group were administered with patients’ written consent when the medicine was not licensed at that time.

Collected data were analyzed using SPSS software (version 24.0; IBM, Armonk, NY). All statistical analyses were performed with a two-tailed test, and a *P*-value lower than 0.05 was considered statistically significant. This study was approved by the Ethics Committee of the NCGM (NCGM-G-002162-00).

## RESULTS

A total of 182 malaria cases were identified. After the exclusion of recrudescence and relapse cases, 169 cases were used for the analysis of basic demography, region of acquisition, travel reason and duration, parasite species, and chemoprophylaxis. Twenty-three of the 169 cases were only followed up after the completion of malaria treatment in hospitals overseas or with stand-by treatment; therefore, 146 cases were used for further analysis. These 146 cases account for one-fifth (20.3%) of the malaria cases reported in Japan and more than half (56.2%) of the cases in Tokyo during the period.^17,18^

### Basic demography.

**Figure 1.**
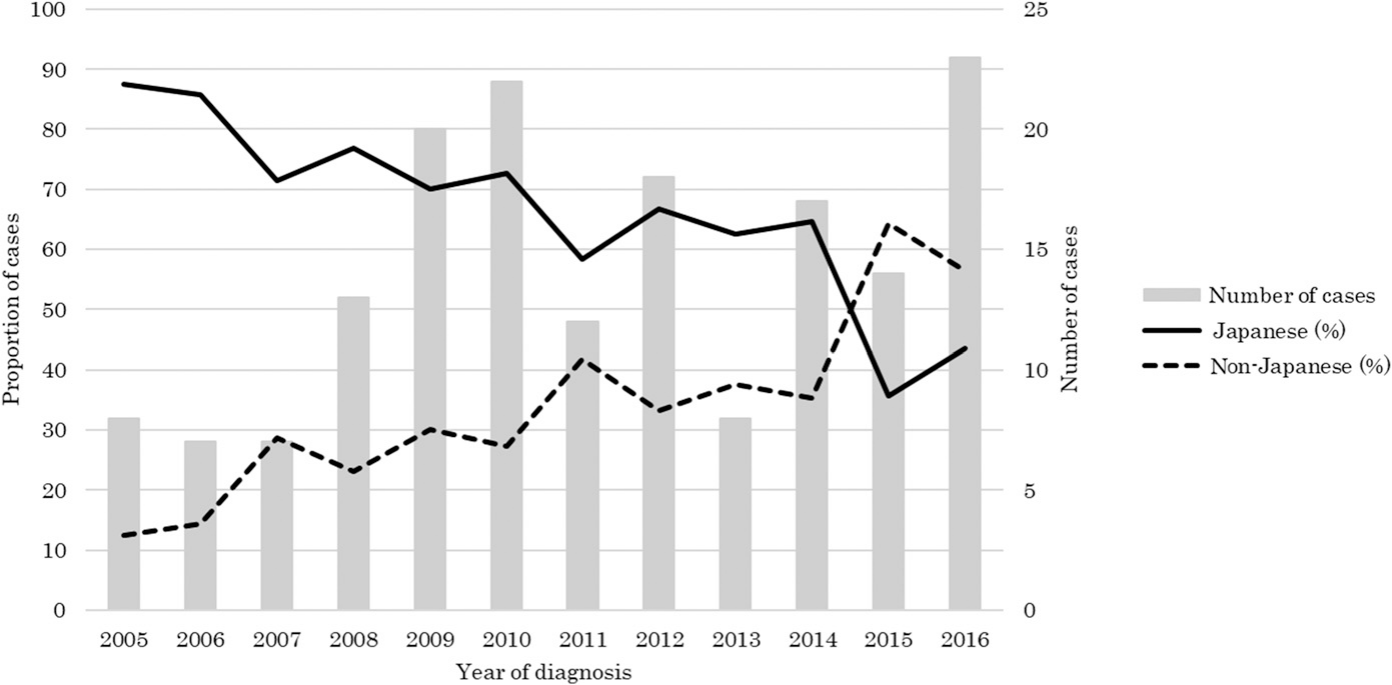
Number of malaria cases observed at the National Center for Global Health and Medicine, by nationality and proportion, among the total cases, 2005–2016.

A total of 79.3% (134/169) of the patients were male and their median age was 34 years (interquartile range [IQR]: 28–43) (Table 1). There were only two pediatric cases aged less than 15 years. Overall, 36.1% (61/169) were non-Japanese patients and their proportion steadily increased and surpassed that of Japanese patients after 2015 (Figure 1). Fifty-three of the patients were citizens of endemic countries: 46 belonged to African countries such as Nigeria (12), Ghana (7), and Cameroon (7), whereas six were from Asia (India [4] and Pakistan [2]), and one was from Oceania (Papua New Guinea).

**Table 1:** Demographic information of the malaria cases at the National Center for Global Health and Medicine by nationality, 2005–2016

	Total	Japanese	Non-Japanese
Number of cases	169	108	61
Age (years) (median, interquartile range)	34 (28–43)	33 (27–43)	37 (30–43)
Gender (male number, %)	134 (79.3)	81 (75.0)	53 (86.9)
Region of acquisition			
Africa	139 (82.2)	86 (79.6)	53 (86.9)
Asia	18 (10.7)	11 (10.2)	7 (11.5)
The Americas	6 (3.6)	6 (5.6)	0
Oceania	6 (3.6)	5 (4.6)	1 (1.6)
Travel reason			
Tourism	27 (16.0)	25 (23.1)	2 (3.3)
Visiting friends and relatives	37 (21.9)	0	37 (60.7)
Business	37 (21.9)	29 (26.9)	8 (13.1)
Missionary	4 (2.4)	4 (3.7)	0
Student	0	0	0
Volunteer	11 (6.5)	10 (9.3)	1 (1.6)
International cooperation activities	33 (19.5)	32 (29.6)	1 (1.6)
Research	6 (3.6)	6 (5.6)	0
Visiting Japan	14 (8.3)	2 (1.9)	12 (19.7)

### Region of acquisition and travel reason and duration.

A total of 82.2% (139/169) of the cases acquired malaria in Africa (Table 1); 58.3%, 27.3%, and 14.4% of these cases were from West, East, and Central Africa, respectively. Asia accounted only for 10.7% (18/169), and most of these patients were from India (10/18). Six cases each were from Oceania (Papua New Guinea) and the Americas. A total of 95.2% (119/125) of the falciparum malaria cases acquired the infection in Africa.

The travel reasons are shown in Table 1. Among Japanese patients, IC activities, business, and tourism were the three most common reasons. In non-Japanese patients, VFR was the most common reason, and most of these patients traveled to African countries. Information on the duration of residency in Japan was available for 29 of the 37 VFR cases, and the median duration of residency was 7 years (IQR: 6–13). The median duration of travel was 33 days (IQR: 18–92), and the median time from return to/arrival in Japan to onset was 9 days (IQR: 3–16).

### Parasite species.

**Figure 2.**
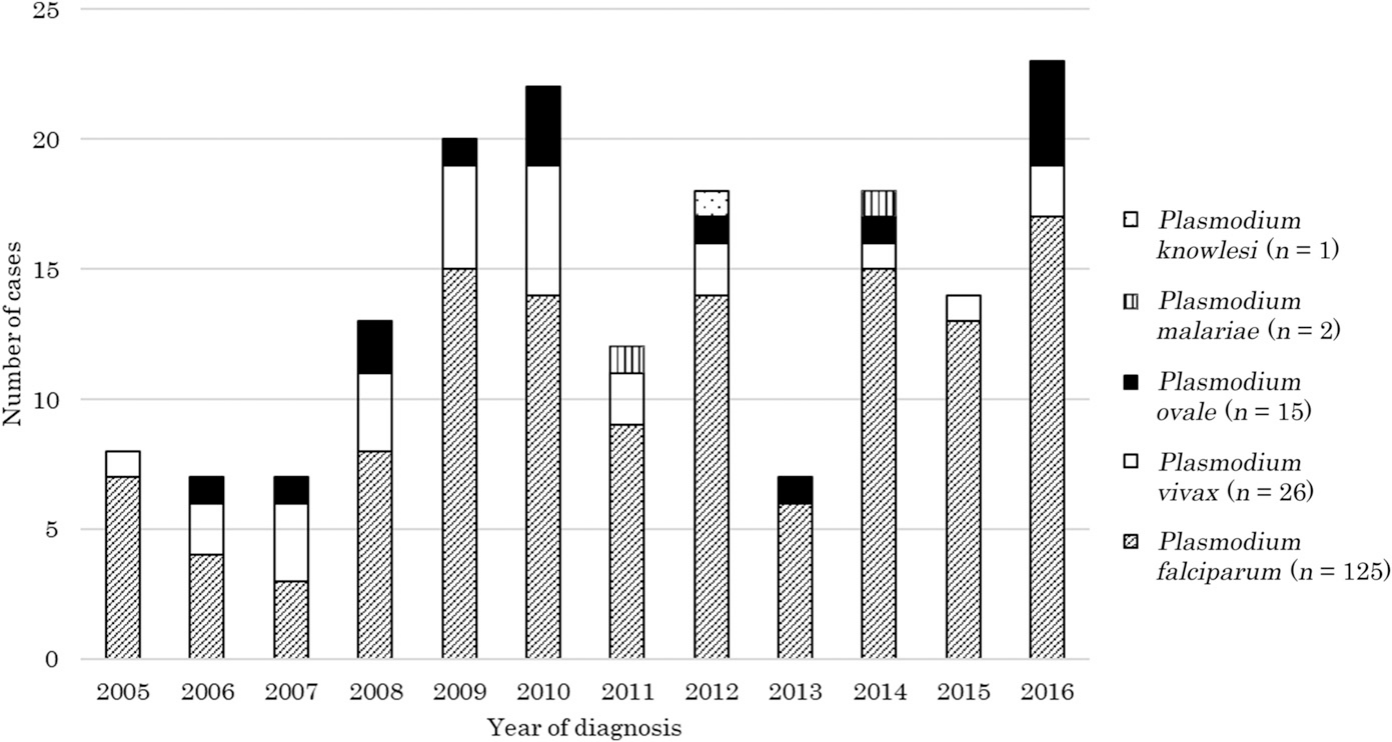
Number of malaria cases observed at the National Center for Global Health and Medicine, by *Plasmodium* species, 2005–2016. The only case of mixed infection (*Plasmodium falciparum* and *Plasmodium ovale*) is counted as *Plasmodium falciparum*.

*Plasmodium falciparum* was the dominant species (125/169 [74.0%]) throughout the study period (Figure 2). Of those who acquired malaria in Africa, 85.5% had *P. falciparum* and 10.1% had *P. ovale* infection. *Plasmodium vivax* accounted for 15.4% (26/169) of the cases but was the dominant species in those who visited Asia, the Americas, and Oceania (78.9%, 83.3%, and 66.7%, respectively).

### Chemoprophylaxis.

Malaria chemoprophylaxis was prescribed for a total of 15.4% (26/169) of the patients (Japanese: 19.4% [21/106]; non-Japanese: 8.2% [5/61]) and for 8.1% (3/37) of the patients on VFR travel. Mefloquine was the most commonly used antimalarial (10/22 [45.5%]). Four Japanese patients claimed to have completed chemoprophylaxis with mefloquine. Three of them stayed in Africa for a long period (9–28 months) and had *P. ovale* infection a few months after their return.

### Diagnosis and treatment.

Rapid diagnostic tests, thin blood smear, and PCR were performed in 94.5% (138/146), 100%, and 100% of the patients. The sensitivities of thin blood smear, compared with those of PCR as a gold standard, were 95.1% (97/102) and 97.1% (43/44) in the falciparum and non-falciparum malaria patients, respectively.

**Figure 3.**
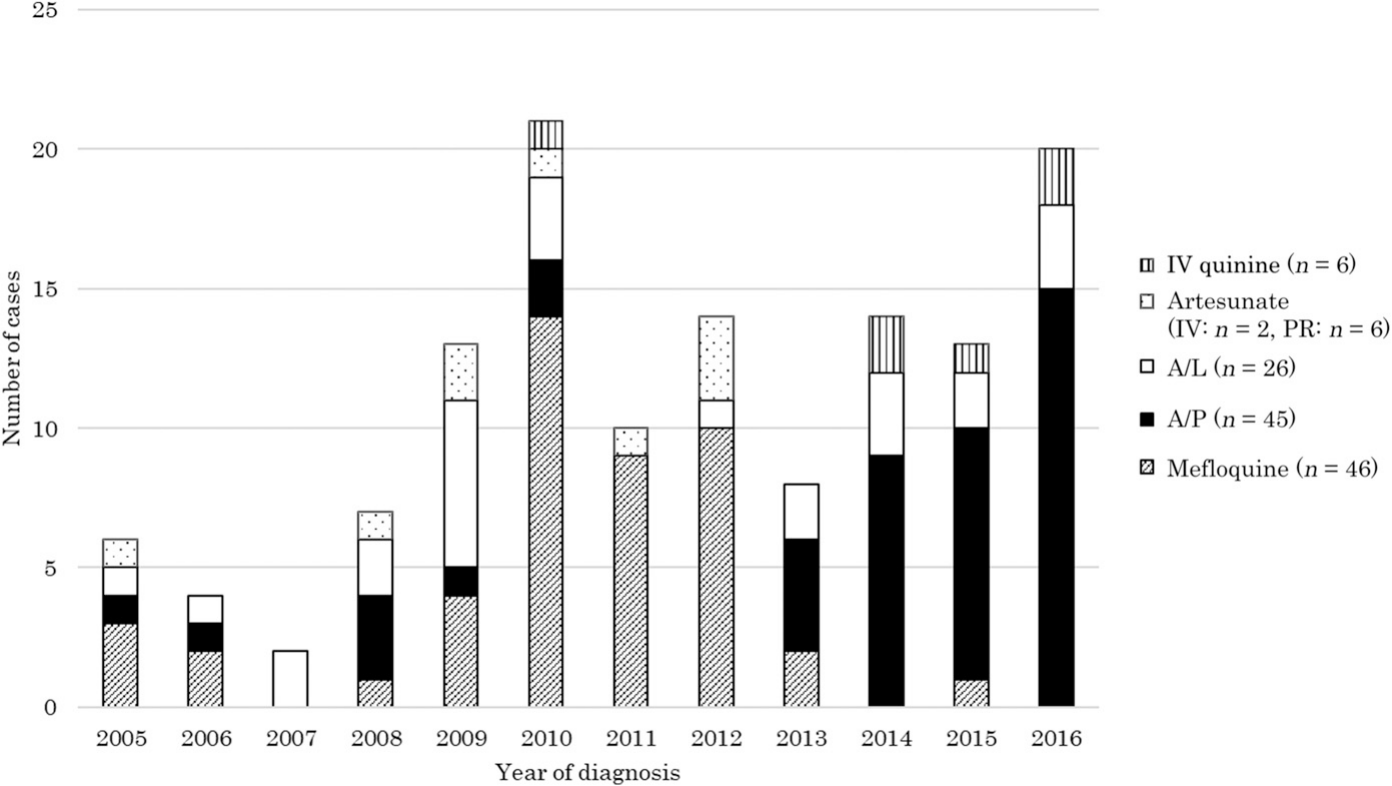
Number of malaria cases treated at the National Center for Global Health and Medicine, by the antimalarial regimen used (quinine, artesunate, artemether/lumefantrine, atovaquone/proguanil, and mefloquine). A/L = artemether/lumefantrine; A/P = atovaquone/proguanil; IV = intravenous; PR = per rectum.

The trend of the first antimalarial regimen is shown in Figure 3. A total of 89.0% (130/146) of the cases were treated only with oral antimalarials. Mefloquine was the most commonly used drug until the early 2010s. After the licensure of A/P in 2013, it was the most commonly used until 2016. Of the 19 severe malaria cases, eight were treated with artesunate, followed most commonly by A/L. Seven patients were treated with injectable quinine followed by oral agents such as A/L, A/P, mefloquine, and clindamycin. Four severe cases were treated only with A/L. All vivax and ovale malaria cases, with the exception of some cases of immediate return to the home country or pregnancy, were successfully treated with primaquine phosphate.

In terms of adverse effects, dizziness due to mefloquine (7/50 [14.0%]) was the most common. Nausea caused by mefloquine (3/50 [6.0%]) or A/L (2/34 [5.9%]) was also observed. Injectable quinine was used in seven patients and five experienced cinchonism of varying severity. Only one patient had related hypoglycemia.

### Outcome.

A total of 74.0% (108/146) of the patients (Japanese: 71/87 [81.6%]; non-Japanese: 37/59 [62.7%]) were hospitalized, and the median duration of hospital stay was 5 days (IQR: 4–8). The duration of hospitalization was longer in the Japanese (seven, IQR: 5–8) than the non-Japanese people (four, IQR: 3–6) (Mann–Whitney *U*-test, *P* < 0.001).

Over the 12-year study period, there were no fatalities. There were four recrudescence cases. All these patients were Japanese nationals weighing more than 65 kg with falciparum malaria of relatively high parasitemia, and treated with A/L. The overall cure rate of A/L was 88.2% (30/34), which is consistent with the results of a recent nationwide survey.^19^ There was one relapse case and the overall relapse rate was 2.4% (1/42). Six Japanese patients developed severe sequelae due to malaria (Table 2).

**Table 2:** Characteristics of the malaria patients who developed severe sequelae

Patient	Country of acquisition	Malaria species	Parasitemia (%)	Disease severity	Antimalarial regimen	Sequelae
54 M, Japanese	Senegal	*Pf*	10.1	Severe	IV artesunate, mefloquine	Post-malaria neurological syndrome
29 F, Japanese	Ghana	*Pf*	12.1	Severe	PR artesunate, IV quinine, A/L	Black water fever
41 M, Nigerian	Nigeria	*Pf*	15.0	Severe	A/L	Black water fever
21 M, Japanese	Kenya	*Pf*	21.7	Severe	Quinine, clindamycin	Neurocognitive dysfunction
56 M, Japanese	Cote d'Ivoire	*Pf*	1.73	Non-severe	A/L	Glomerulonephritis
43 M, Japanese	Zambia	*Pf*	0.035	Severe	A/L	Cardiomyopathy, peripheral gangrene of foot

A/L = artemether/lumefantrine; F = female; M = male; IV = intravenous; PR = per rectum; *Pf* = *Plasmodium falciparum*.

### Severe disease and its risk factors.

There were 19 severe malaria cases, and all of them were caused by *P. falciparum* (18.4%, 19/103). Among the falciparum malaria cases, the disease severity significantly differed by nationality (Japanese: 15/56 [26.8%]; non-Japanese: 4/47 [8.5%]) (χ^2^ test, *P* = 0.017). All these patients were adults and 15 of them were of Japanese nationality. Jaundice (serum bilirubin > 3 mg/dL together with a parasite count > 100,000/µL) was the most common sign of severe falciparum malaria (13/19 [68.4%]), followed by hyperparasitemia (> 10%) (8/19 [42.1%]) and acute kidney injury (serum creatinine > 3 mg/dL or blood urea > 20 mmol/L) (8/19 [42.1%]). Hypoglycemia was not observed. Nine additional patients with falciparum malaria were assessed by attending doctors as having a higher risk of disease progression and treated as severe malaria due to older age, delayed diagnosis, and presence of comorbidities.

Malaria diagnosis on day 5 or later was significantly associated with severe disease among Japanese patients (odds ratio [OR] = 4.1, 95% CI = 1.2–14.3) (Table 3). Age was not significantly associated with severe disease in the Japanese patients (OR = 1.5, 95% CI = 0.4–6.0). There were no severe cases with significant comorbidities.

**Table 3:** Comparison of the severe and non-severe falciparum malaria cases by nationality, 2005–2016

	Total *Pf*, *n* (%)	Severe *Pf*, *n* (%)	Non-severe *Pf*, *n* (%)	Odds ratio	95% CI
Japanese (*n* = 56)					
Age (years)					
< 50	44 (78.6)	11 (73.3)	33 (80.5)	–	–
≧ 50	12 (21.4)	4 (26.7)	8 (19.5)	1.5	0.4–6.0
Gender					
Male	41 (73.2)	11 (73.3)	30 (73.2)	–	–
Female	15 (26.8)	4 (26.7)	11 (26.8)	1.0	0.3–3.8
Day of illness at treatment initiation					
1–4	39 (69.6)	7 (46.7)	32 (78.0)	–	–
≧ 5	17 (30.4)	8 (53.3)	9 (22.0)	4.1	1.2–14.3
Non-Japanese (*n* = 47)					
Age (years)					
< 50	42 (89.4)	4 (100.0)	38 (88.4)	–	–
≧ 50	5 (10.6)	0	5 (11.6)	–	–
Gender					
Male	43 (91.5)	4 (100.0)	39 (90.7)	–	–
Female	4 (8.5)	0	4 (9.3)	–	–
Day of illness at treatment initiation					
1–4	24 (51.1)	1 (25.0)	23 (53.5)	–	–
≧ 5	23 (48.9)	3 (75.0)	20 (46.5)	3.5	0.3–35.8

*Pf* = *Plasmodium falciparum*. A case of mixed infection (*Pf* and *Plasmodium ovale*) is included in this analysis.

### Care-seeking behavior.

Of the 146 treated patients, 136 resided in Japan at the time of malaria diagnosis. More than half of the patients were from Tokyo: 43.4% (59/136) were from the central area (23 special wards of Tokyo) and 14.0% (19/136) from the suburbs. One-third of the patients (45/136 [33.1%]) belonged to the neighboring prefectures. A total of 9.6% (13/136) of these patients were referred from more distant prefectures.

Overall, malaria was diagnosed, and antimalarial therapy was initiated on median day 4 of the illness. The non-falciparum malaria patients received the first antimalarial on median day 6 (IQR: 3–8) of the illness; this was 2 days later than for the falciparum malaria cases (median day 4, IQR: 3–6) (Mann–Whitney *U*-test, *P* = 0.008).

A total of 61.1% (104/144) of the patients visited more than one medical facility before the NCGM. Sixty percent (60/100) of the falciparum malaria patients came through more than one medical facility. The number of medical institutions visited before the NCGM visit correlated with the day of illness when antimalarial treatment was initiated (Spearman’s rank correlation coefficient *r* = 0.363, *P* < 0.01).

## DISCUSSION

This review of malaria cases at a referral hospital in Tokyo highlighted several findings pertaining to imported malaria and ascertained the challenges associated with malaria management in malaria-free countries. There are three significant findings. The first is the dominance of falciparum malaria, which is consistent with the findings of previous studies.^6–8,12^ This dominance is assumed to be due to the decline in the vivax malaria incidence in Asia.^1,20^ According to a British study,^21^ the incidence of vivax malaria imported from travel to India and Pakistan had decreased to one-sixth from 1993 to 2013. The second is the continuous increase in the number of non-Japanese patients. In Japan, the numbers of inbound travelers and migrants have been growing rapidly.^22,23^ Approximately 40% of malaria patients from African countries were from Nigeria and Ghana which have sent the largest African migrant population to our country.^23^ The third is a deficiency of malaria chemoprophylaxis. Only 15.4% of the cases received malaria chemoprophylaxis. The wide use of mefloquine for chemoprophylaxis is also a unique finding of this study. This could be attributed to the fact that A/P was unlicensed until 2013 and the use of doxycycline for malaria chemoprophylaxis is off-label. Atovaquone/proguanil is currently the first choice.

No overall fatality was a favorable outcome. Many studies have pointed to better malaria prognoses in urban areas.^24–26^ This is thought to be explained by the combination of better malaria management and larger semi-immune foreign populations in urban areas. In this study, diagnosis at day 5 or later was a risk factor for severe malaria, but not older age; this could be because of the small number of patients aged 50 years or older. All recrudescence episodes were falciparum malaria cases of Japanese males weighing more than 65 kg and treated with A/L. This is consistent with the results of a recent Swedish study,^27^ although lumefantrine concentration was not measured in our study. The low observed relapse rate is in agreement with the findings of previous Japanese studies.^28^

Overall, malaria was diagnosed rapidly once patients presented to our center. The challenges in malaria management in Japan are its prevention and early diagnosis. Although the ease of availability of anitimalarials should be improved, this may not impact outcomes as significantly as improvements in prevention and diagnosis.

The promotion of pretravel care is pivotal as its success will decrease the overall malaria incidence. Our data on malaria chemoprophylaxis only pertain to those who acquired malaria, and are largely subject to reporting bias; therefore, they should not be overinterpreted. The lack of appropriate chemoprophylaxis, however, is demonstrated. Japanese people with a higher risk of malaria are those who travel to African countries for IC activity, business, research, and school excursions.^12^ It is recommended to educate institutions that send their students and staff to endemic countries, rather than individuals, on the necessity for pretravel care. Visiting friends and relatives travelers are known to be at a higher risk of malaria.^24^ Although their risk of mortality is not as high as that of nonimmune populations,^26,29^ such populations should be targeted too. In Japan, yellow fever vaccination is provided at quarantine centers and some designated hospitals. At quarantine centers, other vaccinations and malaria chemoprophylaxis are not provided. Considering that the endemic areas of malaria and yellow fever overlap considerably, it may be realistic and effective to provide malaria chemoprophylaxis at the time of yellow fever vaccination.

Early diagnosis is important to reduce the severity and fatality associated with malaria. In this study, malaria was diagnosed on median day 4 of the illness and antimalarial therapy was initiated without delay thereafter. No improvements have been achieved in the last two decades.^6^ Malaria diagnosis on day 5 or later was significantly associated with increased disease severity. Delayed diagnosis in non-endemic countries is one of the major risk factors for malaria-related fatality.^24,26,30^ Because the number of hospitals visited before our institute correlated to the day of illness diagnosis, early referral from the medical institutions that first examine the patients is key. However, this is not simple to achieve. Migrant populations tend to be concentrated in urban cities, and two-third of the migrant African population in Japan resides in five prefectures including Tokyo.^23^ Thus, referral hospitals with expertise tend to receive patients with a lower risk of severe malaria. Nonimmune Japanese patients harboring a higher risk, instead, are likely to visit medical facilities in suburban areas in which malaria is rarely observed. Although it is necessary to educate health-care workers in malaria control, it may prove more efficient to inform travelers about which hospitals they should visit and what information they should provide if they experience a fever after returning from a travel.

This study has two strengths. To our understanding, this study represented detailed clinical and laboratory information on the largest number of malaria cases in recent years in Japan. This indicates that the findings of this study provide an overview of malaria in the country. Second, the accuracy of malaria diagnosis was high in our study. All our cases were confirmed by blood microscopy and PCR. The *Plasmodium* species were specified for each case. However, this study has three limitations. First, and most importantly, the unique characteristics of our medical center—a referral hospital for tropical medicine in the capital—may have modified the demography of our malaria patients compared with that of Japan as a whole. Consequently, some of the findings may not be generalizable. Second, our medical center also lays strong emphasis on the provision of good clinical care for foreigners. This could have led to the presence of a higher proportion of non-Japanese patients in our study. Third, as this is a retrospective study comprising a 12-year period, some medical information was missing.

In conclusion, although the demography of malaria cases had not changed considerably, the following findings were highlighted: the dominance of falciparum malaria, increase in the number of non-Japanese cases, late treatment failure after A/L treatment, low relapse rate, delayed malaria diagnosis among severe cases, and improved availability of antimalarials over the study period. For improvements in the clinical management of malaria in settings such as ours, the promotion of pretravel care and early diagnosis and referral are important.
